# Induction of Th17 Lymphocytes and Treg Cells by Monocyte-Derived Dendritic Cells in Patients with Rheumatoid Arthritis and Systemic Lupus Erythematosus

**DOI:** 10.1155/2013/584303

**Published:** 2013-10-29

**Authors:** Lizbeth Estrada-Capetillo, Berenice Hernández-Castro, Adriana Monsiváis-Urenda, Crisol Alvarez-Quiroga, Esther Layseca-Espinosa, Carlos Abud-Mendoza, Lourdes Baranda, Ana Urzainqui, Francisco Sánchez-Madrid, Roberto González-Amaro

**Affiliations:** ^1^Department of Immunology, Facultad de Medicina, UASLP, Avenue V. Carranza 2405, 78210 San Luis Potosí, SLP, Mexico; ^2^Regional Unit of Rheumatology and Osteoporosis, Hospital Central Dr. Ignacio Morones Prieto, San Luis Potosí, SLP, Mexico; ^3^Servicio de Inmunología, Hospital de La Princesa, Universidad Autónoma de Madrid, Instituto de Investigación Sanitaria Princesa, Madrid, Mexico

## Abstract

Dendritic cells (DCs) have a key role in the regulation of immune response. We herein explored, in patients with inflammatory diseases, the role of monocyte derived DC's (mo-DCs) on the generation of Th17 and T regulatory (Treg) lymphocytes. Peripheral blood was obtained from thirty-five patients with rheumatoid arthritis (RA), twelve with systemic lupus erythematosus (SLE), and twenty healthy subjects. Mo-DCs were generated under standard (IL-4/GM-CSF) or tolerogenic (IL-4/GM-CSF plus recombinant P-selectin or PD-1 or IL-10) conditions, and their ability to induce Th17 and Treg lymphocytes was tested. We detected that mo-DCs from patients with RA showed an enhanced release of IL-6 and IL-23 as well as an increased capability to induce Th17 cells. Although mo-DCs from SLE patients also released high levels of IL-6/IL-23, it did not show an increased ability to induce Th17 lymphocytes. In addition, mo-DCs, from patients with RA and SLE generated under the engagement of PSGL-1, showed a defective capability to induce Foxp3+ Treg cells. A similar phenomenon was observed in SLE, when DC's cells were generated under PDL-1 engagement. Our data indicate that DCs from patients with rheumatic inflammatory disease show an aberrant function that may have an important role in the pathogenesis of these conditions.

## 1. Introduction

The defective regulation of the activation and proliferation of auto-reactive T cells that are not deleted in the thymus may result in different autoimmune disorders [1]. Initially, several inflammatory autoimmune diseases were considered to be entirely mediated by Th1 lymphocytes, which mainly synthesize IL-2 and IFN-*γ*. However, in recent years, different studies have demonstrated that Th17 lymphocytes also play an important role in the pathogenesis of these conditions, including multiple sclerosis, psoriasis, inflammatory bowel disease, Hashimoto's thyroiditis, and rheumatoid arthritis [2–8]. In addition, Th17 cells and Th17 cytokines are also involved in the pathogenesis of fibrotic autoimmune diseases as primary biliary cirrhosis (PBC) and systemic sclerosis (SSc) [9]. Th17 lymphocytes are a subset of CD4+ T cells characterized by the synthesis of IL-17A, IL-21, and IL-22, and the expression of IL-23R and CCR6 receptors [10]. Interleukin IL-17A is a proinflammatory cytokine that induces the synthesis of TNF-*α*, IL-1*β*, IL-6, IL-8, GM-CSF, different metalloproteinases (MMP-1, -3, -9), chemokines (CCL2, 3), and PGE2 by different cell types, including fibroblasts, epithelial and endothelial cells, keratinocytes, osteoblasts, and monocyte/macrophages [11–13]. In addition, in patients with rheumatoid arthritis (RA), IL-17 is involved in the destruction of the extracellular matrix and juxtaarticular bone resorption, through the induction of synthesis of RANKL and matrix metalloproteases [14, 15].

The differentiation of human Th17 lymphocytes is induced by the combined action of different cytokines, including IL-1*β*, IL-6, and IL-23 [16], whereas IL-21 promotes the survival and expansion of these cells [17, 18]. In addition, TGF-*β* seems to also participate in the differentiation of Th17 cells, which may have a conventional or a regulatory phenotype [19, 20].

Dendritic cells (DCs) are professional antigen presenting cells that play a key role in the induction and regulation of T cell mediated responses [21]. Two main DC subsets have been characterized in humans, myeloid, and plasmacytoid DCs (mDCs, CD11c^+^, pDCs, and CD11c^−^) [22]. In addition, when monocytes are cultured in the presence of GM-CSF and IL-4, differentiate into a subset of mDCs (monocyte derived DCs or MDDCs or mo-DCs) [23]. DCs express a wide repertoire of membrane receptors, including pattern recognition receptors (PRRs), which upon engagement by their ligands (DAMPS and PAMPs) induce their terminal differentiation and activation [21]. It has been described that a subfamily of PRRs (C-type lectin receptors or CLRs) induce the activation of Syk and CARD9 and preferentially induce the polarization of naive Th0 lymphocytes towards Th17 cells [24–26]. On the other hand, it has also been described that tolerogenic DCs are able to inhibit the differentiation of naïve Th0 lymphocytes, suppressing thus the generation of T cell mediated immune responses [27]. Conversely, tolerogenic DCs are able to induce the generation of T regulatory (Treg) lymphocytes [28]. In this regard, it has been described that different membrane receptors (e.g., PSGL-1 and PDL-1) as well as cytokines (e.g., IL-10) are able to induce the generation of tolerogenic DCs [29, 30]. 

Rheumatoid arthritis (RA) is a systemic inflammatory disease characterized by chronic synovial inflammation, which results in cartilage and bone damage, leading to joint destruction. Different cell types and their mediators are involved in the tissue destructive inflammation seen in this condition, including Th17 lymphocytes [31]. In this regard, mice deficient in IL-17 show a decreased induction of collagen induced arthritis (CIA) [32]. Accordingly, in this animal model of RA, the neutralization of IL-17 reduces joint inflammation, cartilage destruction, and bone erosion [33]. In humans, elevated concentrations of IL-17 have been found in synovial fluid and in peripheral blood of RA patients as well as a high proportion of Th17 lymphocytes in their peripheral blood [34]. In fact, different data indicate that the inflamed rheumatoid synovium is a tissue niche that greatly favors the generation of Th17 cells [35]. Recently, Vaknin-Dembinsky et al. showed that mo-DCs from patients with multiple sclerosis show an enhanced synthesis of IL-23 as well as an increased ability to induce the *in vitro* differentiation of Th17 lymphocytes [36]. However, the precise role of IL-23 and DCs on the induction of Th17 cells in patients with RA has not been fully characterized. 

SLE is an autoimmune, systemic, inflammatory condition with many different immunologic aberrations, including an enhanced synthesis of IL-10 and type I interferon, a diminished function of natural Treg lymphocytes, and an aberrant phenotype and function of DCs [37]. As in the case of RA, different reports indicate that Th17 lymphocytes are involved in the pathogenesis of SLE, including lupus nephritis [38, 39]. We have herein studied the role of immunogenic and tolerogenic DCs on the *in vitro* induction of Th17 cells and Treg lymphocytes in patients with RA and SLE. We detected that mo-DCs from patients with RA show an enhanced release of IL-6/IL-23 and an increased capability to induce Th17 cells. On the other hand, tolerogenic mo-DCs from patients with RA and SLE showed a defective capability to induce the generation of Foxp3+ Treg cells. These results further support the important role of DCs in the pathogenesis of autoimmune diseases, and indicate that it is interesting to comparatively evaluate the generation of effector and regulatory lymphocytes in these conditions [9].

## 2. Materials and Methods

### 2.1. Patients

Thirty-five female patients with RA, with a mean age of 42.3 years, diagnosed according to the criteria of the American College of Rheumatology (ACR) [40] were included in the study. Fifteen patients were untreated at the time of their inclusion in the study, and their mean Disease Activity Score 28 (DAS28) was 4.09. The remaining patients (with a mean DAS28 of 3.45) were under therapy with disease-modifying antirheumatic drugs (DMARDs), receiving mainly low-dose methotrexate (14.9  ±  3.8 mg/week) and sulfasalazine (1.4  ±  0.7 g/day). Twelve female patients with SLE, according to the classification criteria of the American College of Rheumatology [41], were also studied. Their mean age was 33.1 years (range: 16–39), and the mean duration of disease was 19 months. All these patients were not receiving immunosuppressive drugs at the time of the study, and eight of them had active disease, with a MEX-SLEDAI score >3.0 [42]. Three patients were under therapy with low doses of prednisone (10–20 mg/day), and two were receiving hydroxychloroquine (200 mg/day). No patients with renal failure were included. No patients under therapy with biological agents were included in the study. Twenty female healthy individuals with a mean age 35.5 years were included as controls. This study was approved by the local University Ethics Committee and all subjects signed an informed consent.

### 2.2. Cells

Peripheral blood mononuclear cells (PBMC) were isolated from heparinized venous blood by Ficoll-Hypaque density gradient centrifugation (Sigma-Aldrich, St. Louis, MO). Monocytes and CD4^+^ T cells were positively isolated from PBMC by using MicroBeads (Miltenyi Biotec) coupled to mAbs, according to manufacturer's instructions. The purity of the isolated cells was verified by flow cytometry analysis and was always greater than 95%. After CD4^+^ T cells isolation, cells were incubated in complete RPMI culture medium with 2% of fetal calf serum (FCS) until they were used in coculture assays.

### 2.3. Generation of Monocyte-Derived Dendritic Cells (mo-DC)

Monocytes (1 × 10^6^/mL) were plated in RPMI-1640 culture medium supplemented with 10% FCS, 2.0 mM L-glutamine, 100 U/mL penicillin, 100 *μ*g/mL streptomycin (Gibco BRL, Grand Island, NY), 1% sodium pyruvate (Gibco), 1% nonessential amino acids (Hyclone Laboratories, South Logan, UT), and 50 mM 2-mercaptoethanol (Gibco) in the presence of 200 ng/mL rhGM-CSF and 15 ng/mL rhIL-4 (eBiosciences) at 37°C and 5% of CO_2_. Cells were fed on days 2 and 4 by changing one half of the medium and keeping the same concentration of IL-4 and GM-CSF. At day 6, DCs were induced to mature by adding 200 ng/mL of LPS (Sigma-Aldrich). Forty-eight hours after stimulation, supernatants were collected for cytokine measurement. In the case of the induction of tolerogenic dendritic cells, the differentiation of monocytes was carried out in 24-well tissue culture plates previously coated with recombinant human P-selectin (10 *μ*g/mL) or PD1 (2.5 *μ*g/mL) (BioLegend, San Diego, CA) by overnight incubation at 4°C. In other set of experiments, monocytes were induced to differentiate in the presence of IL-10 (40 ng/mL, Biolegend). 

### 2.4. Lymphocyte-DC CoCultures and Induction of Th17 and Treg Differentiation

For the induction of differentiation of Th17 cells, mature mo-DC and autologous CD4^+^ T cells were cocultured in 24-well plates at 1 : 10 ratio in complete Iscove's modified Dulbecco medium (IMDM, Gibco BRL). Plates were previously coated with 10 *μ*g/mL anti-CD3 (eBiosciences) and anti-CD28 (Immunotech) mAbs, and Th17 differentiation was induced by adding rhIL-23 (10 ng/mL, R&D systems, Minneapolis, MN), IL-6 (50 ng/mL), IL-1*β* (10 ng/mL), IL-21 (50 ng/mL), TNF-*α* (10 ng/mL, all from eBiosciences), and anti-IL-4 and anti-IFN-*γ* mAbs (5 *μ*g/mL, BioLegend). Under such conditions, cells were cultured during 5 days at 37°C and 5% of CO_2_, and then the percent of Th1 and Th1/Th17 cells was determined by flow cytometry. In the case of the induction of Th1/Th17 cells, no anti-IFN-*γ* antibody was added. For induction of Treg cell differentiation, DCs generated in the presence of tolerogenic stimuli (IL-10, PD1, P-selectin) were cocultured with autologous CD4^+^ T cells for five days, and then cells were immunostained for CD4, CD25, and Foxp3, and they were analyzed by flow cytometry.

### 2.5. Flow Cytometry Analysis

Myeloid and plasmacytoid dendritic cells were analyzed by using a mixture of mAbs specific for lineage markers combined with HLA-DR, CD11c, BDCA-2, and BDCA-4 mAbs. The following antibodies were used: FITC labeled anti-CD3, anti-CD14, anti-CD16, anti-CD19, and anti-CD56 (eBiosciences); HLA-DR-APC-Cy7, CD11c-PercPCy5.5 (BioLegend), BDCA-2-APC and BDCA-4-APC (Miltenyi Biotec). Cells were analyzed in a FACSAria II flow cytometer (Becton Dickinson, San José, CA) using the FACSDiva software.

For intracellular cytokine analysis, cells were treated for 5 hours with PMA plus Ionomycin (50 ng/mL, and 1 *μ*g/mL, resp., Sigma-Aldrich) and with Brefeldin A (1 *μ*g/mL, Cytofix/Cytoperm kit, eBioscience) for the last 2 hours. Then, cells were stained for the indicated cell surface markers, fixed, permeabilized (Cytofix/Cytoperm kit, eBiosciences), and labeled for IL17 (anti-IL-17-PE, R&D Systems) and IFN-*γ* (anti-IFN-*γ*-FITC, eBiosciences) for 30 min at 4°C. Cells were analyzed in a FACSCalibur flow cytometer (Becton-Dickinson) using the CellQuest Pro software.

Cell surface markers of mo-DCs were analyzed with mAbs directed against CD83, CD86, PSGL-1, PDL-1 (eBiosciences), and HLA-DR (BD Pharmingen). In all analyzes, Fc*γ* receptors were blocked (Fc*γ*R binding inhibitor, eBiosciences) before immunostaining. 

### 2.6. Cytokine*  *Quantification

Levels of IL-23, IL-1*β*, IL-6, IL-22, IL-17A, and IFN-*γ* were quantified in cell-free supernatants using Human ELISA kits (eBiosciences and PEPROTECH) according to manufacturer's instructions. All determinations were performed by duplicate, and plates were analyzed by using a Multiskan ELISA reader. 

### 2.7. Statistical*  *Analyses

Differences between two groups were determined using the Student's *t* test or Mann-Whitney *U* test. One and two way analysis of variance with the proper post hoc analysis was performed when necessary. Data were analyzed using the GraphPad Prism software (GraphPad, San Diego, CA). 

## 3. Results

### 3.1. Peripheral Blood DC Subsets and mo-DCs in Patients with RA and SLE

**Figure 1:**
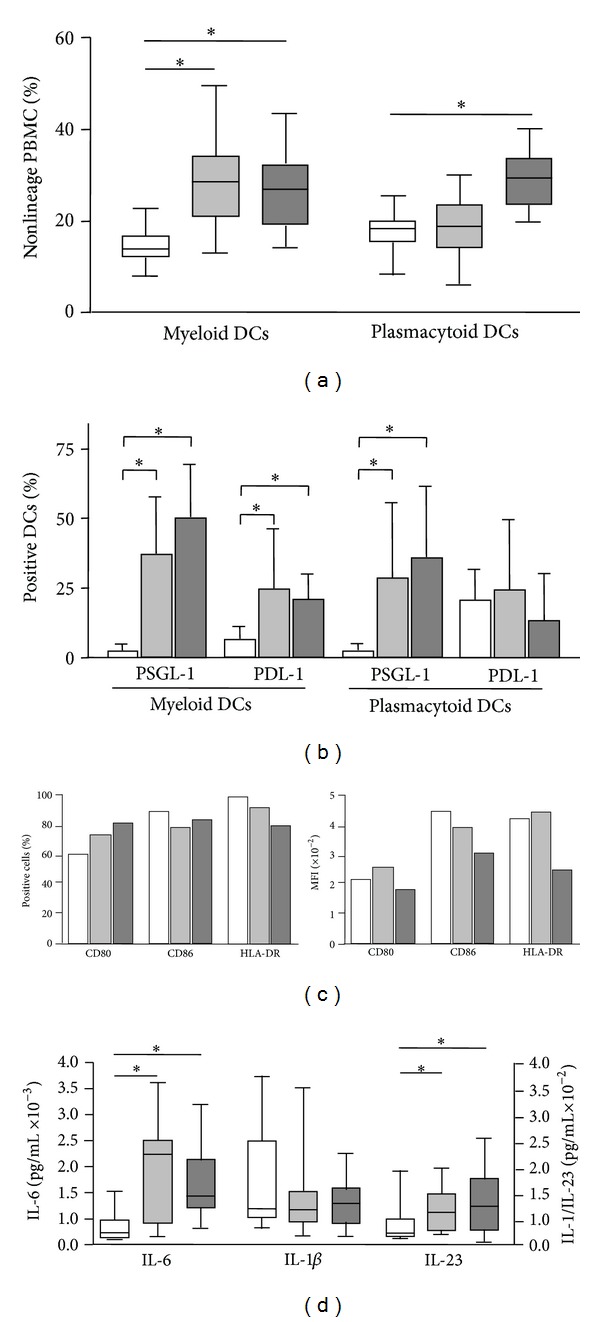
Peripheral blood DCs subsets and mo-DCs in patients with RA and SLE. (a) Myeloid and plasmacytoid DCs levels were determined in freshly isolated peripheral blood mononuclear cells by multiparametric flow cytometry, as stated in materials and methods. Data correspond to the percent of CD11c^+^ BDCA-2+ nonlineage cells (myeloid DCs), and CD11c^−^ BDCA-4+ nonlineage cells (plasmacytoid DCs). (b) The expression of the regulatory receptors PSGL-1 and PD-1 by myeloid and plasmacytoid DCs was analyzed by multiparametric flow cytometry in blood samples from patients with RA and SLE, as stated in materials and methods. (c) mo-DCs were generated *in vitro* by culturing peripheral blood monocytes in the presence of IL-4 and GM-CSF, and their maturation was induced by LPS. Then, cells were immunostained for the indicated molecules, and analyzed by flow cytometry. Data correspond to the percent of positive cells (left panel), and mean fluorescence intensity (MFI, right panel) in cells from representative patients with RA and SLE, and a healthy control. (d) IL-1*β*, IL-6 and IL-23 were quantified in the cell culture supernatants of mo-DCs incubated in the presence of LPS. Median and interquartile range are shown in (a) and (d) and the arithmetic mean and SD in (c). *P < 0.05. In all panels, white boxes correspond to controls, grey light boxes to patients with RA, and grey dark boxes to SLE patients.

We first analyzed the levels of DCs subsets in the peripheral blood from patients and controls. As shown in Figure 1(a), patients with RA and SLE showed an enhanced proportion of CD11c^+^ BDCA-2+ myeloid DCs (*P* < 0.05 in both cases). Furthermore, patients with SLE exhibited higher levels of CD11c^−^ BDCA-4+ plasmacytoid DCs compared to both healthy controls and patients with RA (*P* < 0.05 in both cases). However, no apparent association between disease activity or immunosuppressive therapy and DCs levels was detected in patients with SLE or RA (data not shown). As shown in Figure 1(b), the peripheral blood myeloid DCs from patients with RA and SLE exhibited an enhanced expression of the regulatory receptors PSGL-1 and PDL-1 (*P* < 0.05 in all cases, compared to healthy controls). In the case of plasmacytoid DCs, although the expression of PDL-1 tended to be higher in patients with RA and SLE, no significant differences were observed compared to controls (*P* > 0.05 in both cases, Figure 1(b)). In contrast, the expression of PSGL-1 was significantly higher in plasmacytoid DCs from patients with RA and SLE compared to cells from healthy subjects (*P* < 0.05 in both cases, Figure 1(b)). 

We then generated *in vitro* mo-DCs under standard conditions with IL4/GM-CSF. As shown in Figure 1(c), and as we have previously reported [43], in the case of SLE, these myeloid DCs exhibited a diminished density of expressions of CD80, CD86, and HLA-DR (measured as their MFI). In contrast, the phenotype of mo-DCs was similar in patients with RA and healthy controls. In addition, a similar pattern of PSGL-1 and PDL-1 expression was observed in mo-DCs from the patients included in this study (data not shown). On the other hand, when different cytokines were measured in the cell culture supernatants of mo-DCs, we observed an enhanced release of IL-6 and IL-23 by cells from patients with RA and SLE, compared to healthy individuals (*P* < 0.05 in all cases, Figure 1(d)). In contrast, similar concentrations of IL-1*β* were detected in the cell culture supernatants from patients and controls (*P* > 0.05, Figure 1(d)).

### 3.2. Effect of DCs on the *In Vitro* Differentiation of Th17 Lymphocytes

**Figure 2:**
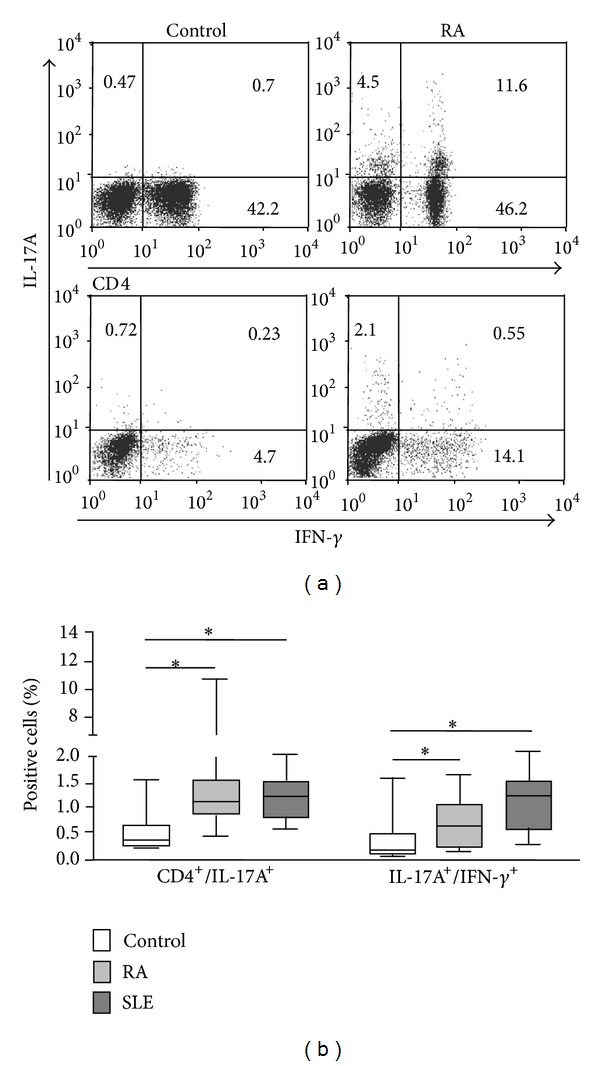
Quantification of Th17 cells in the peripheral blood from patients with RA and SLE. Peripheral blood mononuclear cells from patients with RA and SLE and healthy controls were immunostained for the determination of Th17 (CD4+ IL-17A+) and Th1/Th17 (CD4+ IL-17A+ IFN-*γ*+) lymphocytes, and analyzed by flow cytometry, as stated in materials and methods. Dot plots from cells of a representative patient with RA and a healthy control are shown in (a), and the median and interquartile range of the percent of positive cells in (b). Numbers in quadrants correspond to the percent of events. *P < 0.05.

We first analyzed by flow cytometry the proportion of Th17 cells in the PBMC of healthy controls and patients. As shown in Figure 2, patients with RA and SLE showed enhanced levels of CD4+ IL-17A+ lymphocytes compared to controls (*P* < 0.05 in both cases). In addition, the percentage of Th1/Th17 lymphocytes (defined as CD4+ IL-17A+ IFN-*γ*+) was also higher in patients with RA or SLE compared to healthy subjects (*P* < 0.05 in both cases). Similar results were observed in the case of CD4(−) IL-17+ lymphocytes (*P* < 0.05 in both cases, data not shown). Interestingly, patients with RA under therapy with DMARDs showed significantly lower levels of Th17 cells compared to untreated patients (*P* < 0.05, data not shown). However, no significant correlation between disease activity (DAS28 score) and Th17 levels was detected in these patients (*r* = 0.17,   *P* > 0.05, data not shown). In contrast, a significant correlation between SLEDAI score and Th17 percentages was observed in patients with SLE (*r* = 0.39, *P* < 0.05, data not shown).

**Figure 3:**
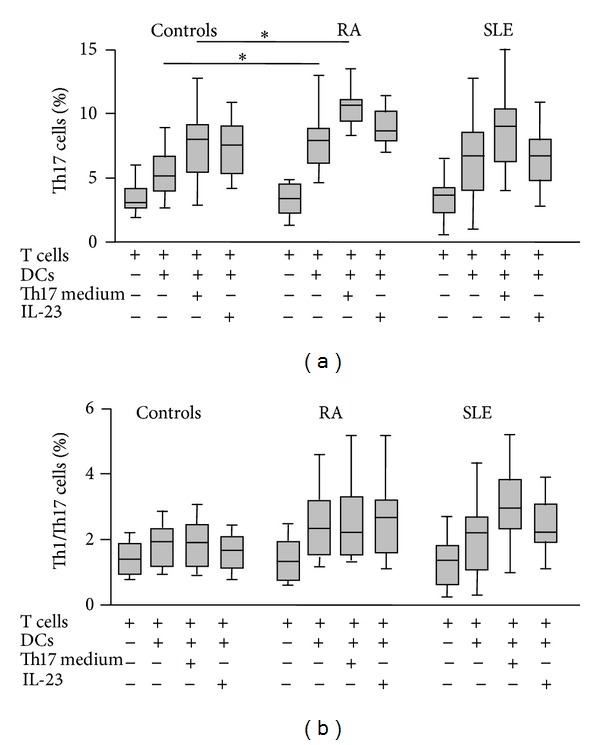
Induction of Th17 and Th1/Th17 lymphocytes by DCs from patients with RA and SLE. Monocyte derived DCs were cocultured with autologous CD4+ cells for five days in the presence or not of a Th17 polarizing medium (IL-1*β*, IL-6, IL-21, and TNF-*α* plus anti-IL-4 and anti-IFN-*γ* blocking antibodies) or IL-23. Then, cells were immunostained for CD4, IL-17A and IFN-*γ* and analyzed by flow cytometry. Data correspond to the median and interquartile range of the percent of positive cells. *P < 0.05.

**Figure 4:**
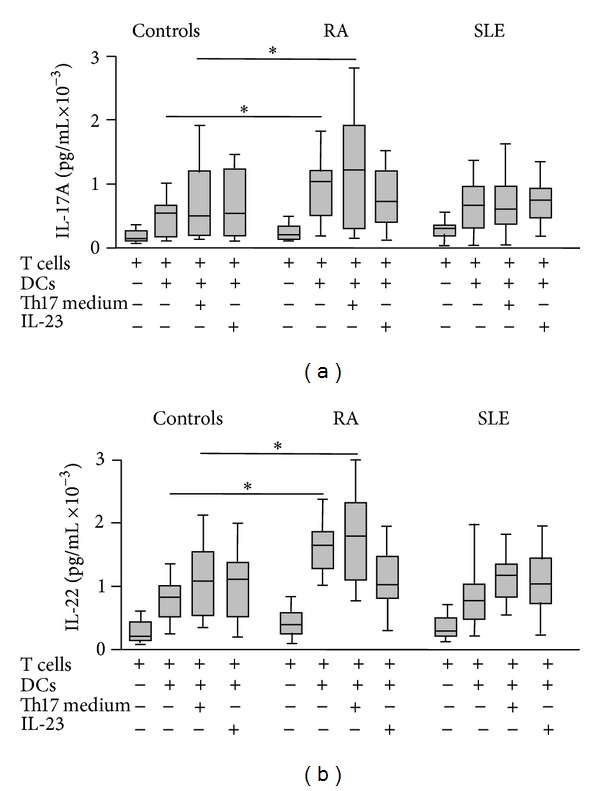
Th17 cytokine synthesis by cocultures of DCs and CD4+ lymphocytes in patients with RA and SLE. IL-17A and IL-22 were quantified in the supernatants of the cell cultures shown in Figure 3. Data correspond to the median and interquartile range of cytokine concentration. *P < 0.05.

When the mo-DCs from patients with RA were cocultured with autologous CD4+ T cells, we detected an enhanced generation of Th17 (CD4+ IL-17A+) lymphocytes (*P* < 0.05 compared to controls, Figure 3(a)). As expected, this phenomenon was enhanced when cells were cultured under Th17 polarizing conditions (addition of IL-1, IL-6, IL-21, and TNF-*α*, with blockade of IL-4 and IFN-*γ*, Figure 3(a)). However, although the addition of IL-23 alone tended to have a similar effect, no significant differences between RA and controls were reached (*P* > 0.05, Figure 3(a)). Furthermore, similar levels of Th17 induction were observed in cells from patients with SLE and controls, in the different conditions tested (*P* > 0.05 in all cases, Figure 3(a)). Finally, although cell cocultures from patients with RA and SLE tended to show an increased generation of Th1/Th17 lymphocytes (CD4+ IL-17A+ IFN-*γ*+), no significant differences were observed when compared to cells from healthy controls (*P* > 0.05 in all cases, Figure 3(b)). In agreement with the above results, we observed a significant enhanced release of Th17 cytokines (IL-17A and IL-22) in the mo-DC/T lymphocyte cocultures from patients with RA (Figure 4). 

### 3.3. Effect of Tolerogenic DCs on the Induction of Treg Lymphocytes

**Figure 5:**
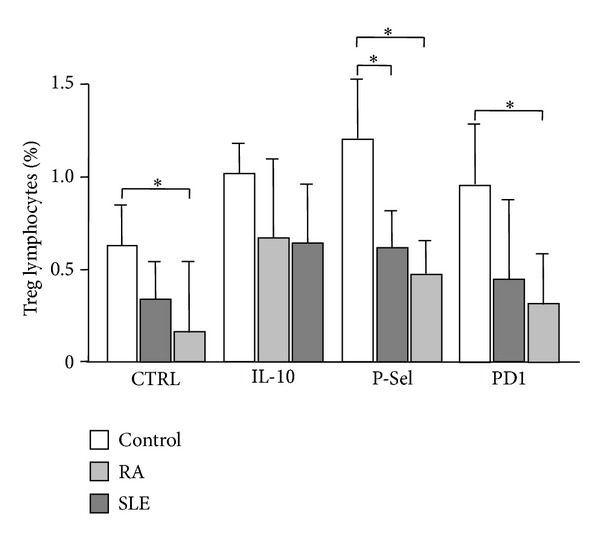
Induction of Treg lymphocytes by mo-DCs from patients with RA and SLE. Monocyte derived DCs generated under tolerizing conditions (PDL-1, PSGL-1 engagement, or IL-10 addition) or not, were cocultured with autologous CD4+ lymphocytes for five days. Then, the percent of CD4+ CD25+ Foxp3+ lymphocytes was determined by flow cytometry. Data correspond to the median and interquartile range of the percent of positive cells. *P < 0.05.

An additional analysis of the functional status of mo-DCs in SLE and RA was performed, by measuring their capability to induce Treg lymphocytes. When mo-DCs were generated under standard conditions (IL-4/GM-CSF), those derived from SLE patients showed a diminished capability to induce the generation of autologous CD4+ CD25+ Foxp3+ Treg cells (*P* < 0.05 compared to controls, Figure 5). Interestingly, when mo-DCs were generated under the engagement of the tolerogenic receptor PSGL-1 (by the addition of exogenous P-selectin), a diminished capability to promote the generation of Treg lymphocytes was observed in patients with RA and SLE (*P* < 0.05 compared to controls, in both cases, Figure 5). A similar phenomenon was observed in the case of mo-DCs from patients with SLE, when they were generated in the presence of recombinant PD-1 (*P* < 0.05 compared to controls, Figure 5). Although mo-DCs from patients with RA generated under PDL-1 engagement tended to show a diminished ability to induce Treg cells, no significant difference was reached in this case (*P* > 0.05, Figure 5). 

## 4. Discussion

 Different data indicate that in the complex pathogenesis of RA and SLE, DCs as well as Th17 and Treg lymphocytes have an important role [8, 31, 36]. In this work, we first analyzed the levels of Th17 lymphocytes and the two main subsets of peripheral blood DCs in samples from these patients and healthy controls. In agreement with previous reports [12, 44, 45], and as has been observed in other conditions such as PBC or SSc [9], we detected enhanced levels of Th17 lymphocytes in the peripheral blood from patients with RA and SLE. However, it is of interest that other authors have not observed increased numbers of these T helper cells in patients with RA [43]. It is feasible that these apparent contradictory results could be due to differences in the therapy, time of disease evolution, level of disease activity or genetic background of patients. In this regard, we have detected that those patients under therapy with DMARDs exhibit lower percentages of Th17 cells than those in untreated patients. In addition, we found a significant association between disease activity and Th17 cell levels in patients with SLE. However, in patients with RA this correlation was not detected. All these data indicate that it would be interesting to perform a longitudinal study of Th17 cell levels in a cohort of patients with RA and SLE. 

 In order to further explore the role of DCs in the induction of Th17 lymphocytes in patients with RA and SLE, we performed *in vitro* experiments with mo-DCs. In this regard, it is worth mentioning that although this type of DCs is not detected in the peripheral blood of healthy subjects, different evidences indicate that this subset of antigen presenting cells is generated *in vivo* [46]. Our results indicate that the mo-DCs from patients with RA have an increased capability to induce the *in vitro* differentiation of Th17 lymphocytes as well as an enhanced potential to release cytokines that allow the generation of these cells. These data further support the important role of DCs in the pathogenesis of RA and indicate that this functional feature is preserved during their *in vitro* generation from peripheral blood monocytes. In addition, our results also support that the inflamed rheumatoid synovium is a privileged site for the generation of Th17 cells [35]. Since this enhanced capability of mo-DCs to induce Th17 lymphocytes was not observed in patients with SLE, our data indicate that, under our experimental conditions, the overproduction of IL6/IL-21/IL-23 by DCs is not enough to increase the differentiation of Th17 lymphocytes. In this regard, it is of interest that we have corroborated [47] that mo-DCs from patients with SLE show a defective expression of HLA-DR and the costimulatory molecule CD86. Thus, it is feasible that this defective phenotype of SLE mo-DCs accounts, at least in part, for their diminished ability to induce Th17 cells, compared to cells from patients with RA.

 We have detected that the levels of peripheral blood mDCs are enhanced in patients with RA, and this finding is in disagreement with a previous report [48]. As in the case of Th17 cells, it is feasible that different factors could account for this apparent contradictory results. In this regard, it seems evident that the levels of DCs in peripheral blood are strongly influenced by their rates of generation in bone marrow and their extravasation to different tissues, phenomena that are not easy to analyze *in vivo*, in human beings. Thus, we consider that it would be easy and interesting to perform a prospective study on the levels of the different DC subsets in the peripheral blood in patients with RA and SLE.

 Patients with RA and SLE show a defective function of nTreg cells, and abnormal numbers and proportions of Treg lymphocytes have been reported in these patients [49–51]. Since DCs have an important role in the generation of induced Treg (iTreg) cells, we decided to generate *in vitro* tolerogenic DCs and test their ability to trigger the differentiation of these regulatory lymphocytes. We have detected that upon engagement of the tolerogenic receptor PSGL-1, the mo-DCs from patients with RA and SLE showed a defective capability to induce the generation of iTreg cells. We consider that this is an interesting finding since further supports the important role of DCs in the defective immune-regulation observed in these patients. In addition, it is also of interest that this aberrant functional feature is observed after their *in vitro* differentiation from peripheral blood monocytes. In this regard, it would be of interest to determine whether this type of mo-DCs are generated *in vivo*, in patients with RA and SLE, in their diseased, and to explore their possible contribution to the defective regulation of the inflammatory phenomenon. The possible cause of the enhanced expression of PSGL-1 and PDL-1 by the mo-DCs from these patients also remains as an interesting point to be explored.

 Since it has been previously reported that patients with SLE and RA show an enhanced synthesis of IL-10 [36, 52], it is of interest that this cytokine had showed a tolerogenic effect on mo-DCs that was not significantly different from that found in patients compared to controls. Thus, this finding suggests that although the overproduction of IL-10 may contribute to the pathogenesis of SLE and RA [37, 52]. It is also feasible that the enhanced synthesis of this cytokine could favour the generation of tolerogenic DCs *in vivo* in these patients. In contrast, our findings indicate that the other tolerogenic stimulus employed in this study (PD-1) is not able to exert a significant effect on the mo-DCs from patients with SLE. This finding further suggests that the defective immune-regulatory function observed in patients with SLE is the result of many different factors, including the diminished activity of several tolerogenic receptors expressed by DCs.

## 5. Conclusions

Our findings strongly suggest that mo-DCs may significantly contribute to the enhanced generation of Th17 lymphocytes, and the diminished number and activity of Treg cells observed in patients with RA and SLE. Thus, the possible functional role of this subset of DCs *in vivo*, in the pathogenesis of autoimmune inflammatory conditions, is an interesting point to be further explored.
